# Integrated spatial analysis of gene mutation and gene expression for understanding tumor diversity in formalin-fixed paraffin-embedded lung adenocarcinoma

**DOI:** 10.3389/fonc.2022.936190

**Published:** 2022-11-24

**Authors:** Miki Yamazaki, Masahito Hosokawa, Hiroko Matsunaga, Koji Arikawa, Kazuya Takamochi, Kenji Suzuki, Takuo Hayashi, Hideki Kambara, Haruko Takeyama

**Affiliations:** ^1^ Department of Life Science and Medical Bioscience, Waseda University, Tokyo, Japan; ^2^ Computational Bio Big-Data Open Innovation Laboratory, National Institute of Advanced Industrial Science and Technology, Tokyo, Japan; ^3^ Research Organization for Nano and Life Innovation, Waseda University, Tokyo, Japan; ^4^ Institute for Advanced Research of Biosystem Dynamics, Waseda Research Institute for Science and Engineering, Waseda University, Tokyo, Japan; ^5^ Department of Thoracic Surgery, Juntendo University School of Medicine, Tokyo, Japan; ^6^ Department of Human Pathology, Graduate School of Medicine, Juntendo University, Tokyo, Japan; ^7^ Frontier BioSystems Inc., Tokyo, Japan

**Keywords:** spatial transcriptome, non-small cell lung cancer (NSCLC), tyrosine-kinase inhibitors (TKIs), drug resistance, formalin-fixed paraffin-embedded specimens, tumor microenvironment, intratumoral heterogeneity (ITH), cancer therapy

## Abstract

**Introduction:**

A deeper understanding of intratumoral heterogeneity is essential for prognosis prediction or accurate treatment plan decisions in clinical practice. However, due to the cross-links and degradation of biomolecules within formalin-fixed paraffin-embedded (FFPE) specimens, it is challenging to analyze them. In this study, we aimed to optimize the simultaneous extraction of mRNA and DNA from microdissected FFPE tissues (φ = 100 µm) and apply the method to analyze tumor diversity in lung adenocarcinoma before and after erlotinib administration.

**Method:**

Two magnetic beads were used for the simultaneous extraction of mRNA and DNA. The decross-linking conditions were evaluated for gene mutation and gene expression analyses of microdissected FFPE tissues. Lung lymph nodes before treatment and lung adenocarcinoma after erlotinib administration were collected from the same patient and were preserved as FFPE specimens for 4 years. Gene expression and gene mutations between histologically classified regions of lung adenocarcinoma (pre-treatment tumor in lung lymph node biopsies and post-treatment tumor, normal lung, tumor stroma, and remission stroma, in resected lung tissue) were compared in a microdissection-based approach.

**Results:**

Using the optimized simultaneous extraction of DNA and mRNA and whole-genome amplification, we detected approximately 4,000–10,000 expressed genes and the epidermal growth factor receptor (EGFR) driver gene mutations from microdissected FFPE tissues. We found the differences in the highly expressed cancer-associated genes and the positive rate of EGFR exon 19 deletions among the tumor before and after treatment and tumor stroma, even though they were collected from tumors of the same patient or close regions of the same specimen.

**Conclusion:**

Our integrated spatial analysis method would be applied to various FFPE pathology specimens providing area-specific gene expression and gene mutation information.

## Introduction

Lung cancer is the leading cause of cancer-related deaths worldwide. Among them, non-small cell lung cancer (NSCLC) accounts for approximately 80% of all lung cancers (1). The epidermal growth factor receptor tyrosine kinase inhibitors (EGFR-TKI) show a high response rate in NSCLC patients with driver gene mutations (2). However, the problem is that most patients eventually develop drug resistance (3). The mechanisms of resistance acquisition to EGFR-TKI are various, including EGFR somatic mutations (T790M and V843I), amplification of receptor genes alternative for EGFR (MET and HER2), mutations in downstream genes of the EGFR pathway (PIK3CA and BRAF), and phenotype changes associated with epithelial-mesenchymal transition (EMT) (4). Several sources of resistance acquisition have been attributed to intratumoral heterogeneity of gene mutations, gene expression or interactions between tumor cells, and stroma in the tumor microenvironment (5). Therefore, understanding cellular diversity before and after drug treatment is essential for elucidating drug resistance mechanisms and determining prognostic factors in NSCLC.

Tumors have a unique tumor microenvironment different from normal tissues, consisting of tumor stroma that promotes the survival, proliferation, and invasion of tumor cells (6–9). The single-cell RNA-seq (scRNA-seq) has revealed intratumor cellular diversity, including 14 constituent cell types in NSCLC and their involvement in immune status (10), as well as cell–cell interactions correlated with tumor phenotypes in human melanoma (11). In recent single-cell scRNA-seq studies, single-cell barcode technology, droplet-based high-throughput platforms (12), and Chromium single-cell gene expression (10× Genomics) automate the process from single-cell isolation to sequence library preparation, making it possible to analyze thousands of cells at once. However, scRNA-seq does not provide spatial information for specific gene expression. In a study comparing the molecular characteristics of four different regions in the histology and stage of lung adenocarcinoma, tumor mutation burden, gene expression profiles, and DNA chemical modifications differed by region even within the same tissue (13), thus indicating the importance of transcriptome and genome analysis integrated with tissue spatial information.

Spatial transcriptome (ST) analysis is a method of assigning gene expression data to a spatial location within the tissue (14) and has been used to investigate intratumoral heterogeneity (15, 16). Various ST methods have been reported, such as the tissue microdissection-based approach (17, 18), spatial barcode-based approach (19–21), and imaging-based approach (22). However, a method for simultaneously extracting DNA and RNA from specific tissue areas has not yet been integrated with ST and gene mutation analyses. Pathological tissues collected in clinical practice are generally preserved as formalin-fixed paraffin-embedded (FFPE) specimens. In FFPE specimens, methylene cross-links are formed between nucleic acids and proteins, making it more challenging to extract and amplify the nucleic acids. If the integrated spatial analysis of gene mutation and expression of FFPE specimens becomes possible, we will be able to obtain the data from the estimated 500 million FFPE specimens linked to clinical information stored worldwide (23). It would elucidate tumor heterogeneity for understanding new molecular mechanisms and the discovery of biomarkers in specific areas in tumor tissues.

Here, we developed a method for simultaneous mRNA and DNA extraction from the microdissected FFPE tissue (100 μm in diameter and 10 μm in thickness) and established a technique for simultaneous analysis of ST and gene mutation in pathological tissues. We investigated the gene expression profile and the diversity of gene mutations before and after the administration of EGFR-TKI at the tumor microenvironment level. Using the microdissection punching system (24), we collected microdissected tissues of five regions from the 4-year preserved FFPE lung adenocarcinoma specimens. Different expression levels of NSCLC tumor markers, fibroblast markers, and myofibroblast markers were observed in the adjacent tumor, tumor stroma, and remission stromal regions on the same tissue section. The microdissected tissues positive for EGFR exon 19 deletion (a driver gene mutation of EGFR-TKI) was scattered in the same tumor site. Our validation suggested that the integrated ST and gene mutation analysis could be applied to valuable FFPE pathological tissue specimens stored in medical institutions for a long time and contribute to revealing tumor heterogeneities.

## Materials and methods

### Sample acquisition

The tumor used for this analysis was provided by the Department of Human Pathology, Juntendo University School of Medicine. All tissue samples were fixed in 10% neutral-buffered formalin for 24h at room temperature, embedded in paraffin after routine processing. The biopsy sample was examined for EGFR exon 19 deletion with cobas^®^ EGFR Mutation Test v2 (25) and tested positive. The procedures involving human participants were approved by the regional ethical committee at Juntendo University School of Medicine (no. 2018090). The study conformed with the 1964 Helsinki declaration and its amendments or comparable ethical standards.

All mice (ICR, male, > 10 months old, Tokyo Laboratory Animals Science Co. Ltd., Tokyo, Japan) used for the optimization of mRNA and DNA extraction and whole-genome amplification (WGA) methods were treated according to the protocols approved by the Committee for Animal Experimentation of the School of Science and Engineering at Waseda University (No. 2017-A056 and No. 2018-A067) and in accordance with the law (No. 105) passed by and notification (No. 6) of the Japanese Government. Sliced FFPE tissues were prepared as described previously (26).

### Tissue sectioning, staining, and imaging

FFPE material was sectioned at 10 μm with a microtome (Yamato, Saitama, Japan), and then, the tissue sections were transferred on an LMD film II (SECTION-LAB, Hiroshima, Japan). We conducted de-paraffinization of the tissue section soaking in Hemoclear, and the tissue section was stained using hematoxylin and eosin. Whole images of tissue sections were captured as described previously (27). According to the tiled images of tissue, we had morphologically classified the anatomical areas of interest and collated them with gene expression data individually indexed in the RNA-seq library.

### Total RNA and DNA extraction from the tissue section

Total RNA and DNA were extracted from FFPE specimens of lung adenocarcinoma using RNeasy mini kit (QIAGEN, Hilden, Germany) or DNeasy blood and tissue kit (QIAGEN) according to the manufacturer’s protocol and stored at -80°C. The RNA integrity number equivalent (RINe) and DNA integrity number (DIN) were measured using the Tapestation 4200 (Agilent, Tokyo, Japan). The RNA concentration was measured using Qubit (Thermo Fisher Scientific, Waltham, MA, USA).

### Tissue microdissection with the tissue microdissection punching system

These steps have previously been reported (24). Briefly, the microdissected tissue with a diameter of 100 μm was punched from the tissue section using the tissue microdissection punching system with the hollow punching needle (Frontier Biosystems, Tokyo, Japan). The collected position was manually selected while observing the tissue with a microscope and monitor, and the punching operation was carried out continuously.

### Simultaneous extraction of mRNA and DNA from the microdissected tissue and library preparation

For mRNA extraction from tissue microdissections, we used the method using oligo dT magnetic beads as previously reported (27). In short, tissues were lysed using Proteinase K followed by poly (A) RNA purification using oligo dT magnetic beads. For FFPE specimens, tissue lysis was followed by decross-linking treatment at 85°C for 5 min. The purified mRNA was directly processed according to Smart-seq2 (28). DNA was purified using 1.5 times the amount of SPRI beads (Beckman coulter, Brea CA, USA) from the supernatant after the annealing poly (A) RNA with oligo dT. For FFPE specimens, decross-linking treatment at 90°C for 5 min was performed before DNA purification. The purified DNA was directly used for WGA using the GenomePlex^®^ Complete WGA kit (Sigma Aldrich, St. Louis, MO, USA).

### Detection of EGFR exon 19 deletion

The EGFR exon 19 sequence was amplified by polymerase chain reaction (PCR), using specific primers (**Supplementary Tables S1** and **S2**). One microliter of whole genome amplified DNA was mixed with 0.2 μl of forward and reverse PCR primers, 5 μl of PyroMark PCR Master Mix (QIAGEN) and 3.6 μl of nuclease-free water. The reaction mixture was incubated at 95°C for 15 min, followed by 40 cycle of 94°C for 30 sec, 62°C for 30 sec, and 72°C for 30 sec. It was ended with a final extension at 72°C for 10 min. Library size distribution was checked on DNA 1000 chip (Agilent Bioanalyzer) with undiluted PCR amplicons.

### Sequencing and data analysis

Sequencing and data analysis was carried out as described previously (27). Amplified cDNA (0.25 ng) was used to prepare the sequencing library using the Nextera XT DNA library prep kit. Paired-end sequencing was performed on the MiSeq, with 75 bases for read 1 (R1) and 75 bases for read 2 (R2). We trimmed the adapter sequences in all the sequence reads using flexbar 3.3.0. The trimmed sequence reads were aligned to the Ensembl human reference genome (GRCh38 ver. 96) for human tumor samples, including the ERCC sequences using hisat 2 2.1.0 with the default parameters. The gene expression levels, given as transcript per million (TPM), were calculated using samtools 1.7 with a transcriptome reference obtained from Ensembl. The detection of differentially expressed genes was conducted using edgeR normalized count.

### Quantification and statistical analysis

Statistical analyses were performed using the Rstudio (v2021.09.2+382) within R version 4.1.2. All statistical details, including the statistical tests, a represented number of samples, dispersion, and precision measures, can be found in the results and legends of the respective figures when noted appropriately.

## Results

### Overview of simultaneous analysis of spatial transcriptomics and gene mutation with the lung adenocarcinoma before and after EGFR-TKI treatment

**Figure 1:**
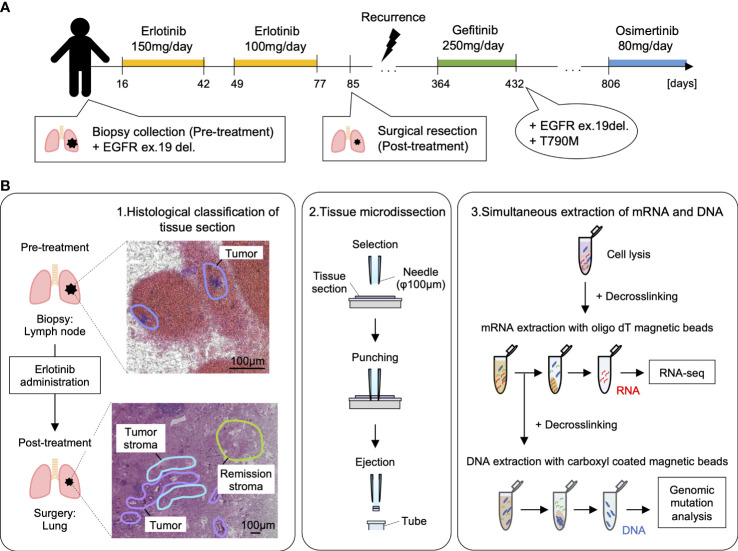
Overview of spatial transcriptome and gene mutation analysis with lung adenocarcinoma before and after erlotinib administration. **(A)** The medical history of the patients analyzed in this study. By punch biopsy, lung lymph nodes were collected before erlotinib administration and used for gene mutation testing. The patient was treated with erlotinib 150 mg/day for 26 days, withdrawn due to side effects such as anemia, and then started on erlotinib 100 mg/day after symptoms improved. The lung tumor was surgically removed about 70 days after starting the medication. After that, the disease recurred, and the patient was treated with gefitinib 250 mg/day, but EGFR ex19del and T790M were detected, so the dosage was changed to osimertinib 80 mg/day on 9 October 2019, and he is alive now. **(B)** Workflow of simultaneous analysis of transcriptome and gene mutation from tissue microdissections in lung adenocarcinoma. 1: Morphological classification of adenocarcinoma by the experienced pathologist. 2: Micro-area with a diameter of 100 µm of the tumor, tumor stroma, remission stroma, and normal lung are collected from lung adenocarcinoma after erlotinib administration by microdissection punching system. In the pre-treatment lung lymph node, the micro-area in the tumor is collected. 3: The tissue microdissections lysed with proteinase K followed by the mRNA extraction with oligo dT magnetic beads. Extracted mRNA is decross-linked by incubation at 85 °C for 5 min and used for RNA-seq. After mRNA purification, DNA was extracted with carboxyl-coated magnetic beads from the supernatant. Extracted DNA was decross-linked by incubation at 90°C for 5 min and used for the detection of EGFR driver gene mutation.

We studied lung lymph node biopsies before erlotinib administration and resected lung adenocarcinoma after administration in one identical patient. The patient was treated with erlotinib as a neoadjuvant therapy because of EGFR exon19 deletion (ex19del)-positive in the gene mutation test of the lung lymph node biopsy (**Figure 1A**). The patient underwent surgical resection of the tumor site after erlotinib administration but relapsed approximately 9 months later, and the treatment was switched to the administration of gefitinib instead. Approximately 2 months later, resistance mutation (T790M) of the first-generation molecular target drugs (erlotinib and gefitinib) was detected. The treatment was then changed to the second-generation molecular target drug (Osimertinib), and the patient is currently alive. The lung specimens before and after erlotinib administration were collected on 25 July 2017 and 18 October 2017 and, subsequently, FFPE and stored at room temperature for approximately 4 years. The DIN and RINe showed a state of progressive degradation in DNA and DNA extracted from a lung tissue section after erlotinib administration (RINe: 1.1 ± 0.1, DIN: 5.9 ± 0.3) (**Supplementary Tables S3**, **S4**) (29).

**Figure 2:**
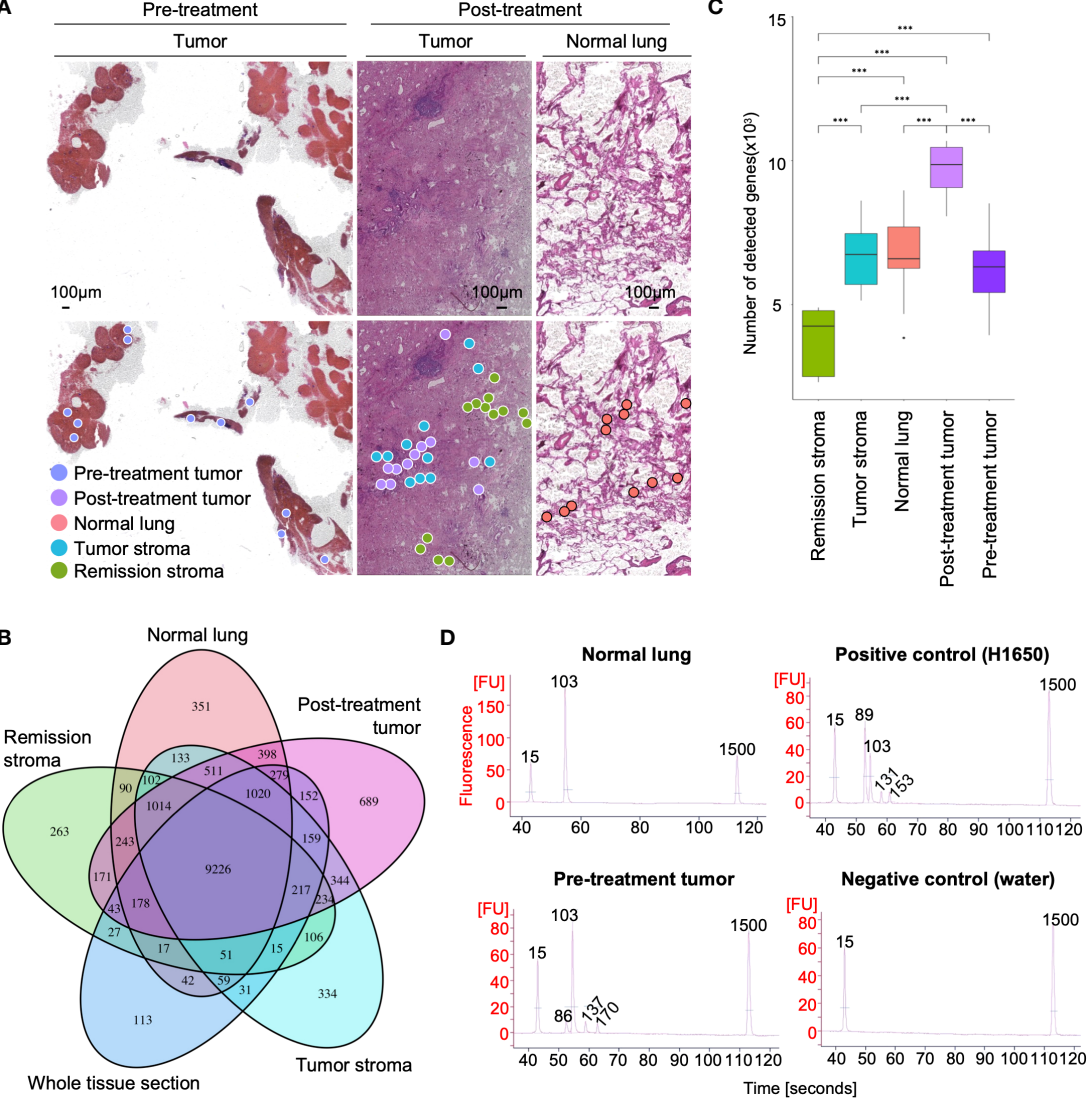
RNA-seq and EGFR exon 19 deletion detection from microdissected FFPE tissues of lung adenocarcinoma. **(A)** Spatial distribution of sampling locations. Left: tumor in pre-treatment lung lymph node; middle: tumor, tumor stroma, and remission stroma in post-treatment lung adenocarcinoma; right: normal lung in post-treatment lung adenocarcinoma. **(B)** Venn diagram of the genes detected from tissue microdissections or the whole tissue section of post-treatment lung adenocarcinoma. The genes detected from tissue microdissections were merged by histological classification (post-treatment tumor, normal lung, tumor stroma, and stroma in remission). **(C)** The number of genes detected from tissue microdissections. Comparison of the number of detected genes between areas with different histological classifications. Student’s t-test, ***P < 0.001. **(D)** Results of EGFR ex19del detection. The EGFR ex19del region was PCR amplified, and Bioanalyzer DNA1000 measured the length of the PCR amplified product. The 15 bp and 1500 bp peaks indicate lower and upper markers, respectively. The peak at 86–89 bp is deletion positive, and the peak at 103 bp is wild type (The peaks at 131–170 bp represent the by-products generated by PCR amplification).

The specimens before and after erlotinib administration were thinly sectioned to a thickness of 10 µm, and pathologists analyzed HE-stained histological images to diagnose histological regions (**Supplementary Figure S1**). We used these annotations for classification during the analysis of differentially expressed genes. Tissue microdissections (100 μm in diameter) were collected from the histologically classified regions (pre-treatment tumor in lung lymph node biopsies and post-treatment tumor, normal lung, tumor stroma, and remission stroma, in resected lung tissue). DNA and RNA were continuously extracted from each tissue microdissection (**Figures 1B**, **2A** and **Table 1**). The tissue microdissections were decross-linked using Proteinase K and heat treated. The mRNA was then purified using oligo dT magnetic beads (27). DNA was purified from the supernatant after mRNA separation using AMPure beads. The purified mRNA was used for RNA-seq, and the purified DNA was used for mutation detection.

**Table 1:** Description of processed samples.

Collection	Tissue	Gene mutation testing	Treatment	Histological classification	Replicate
Biopsy	Lung lymph	EGFR exon19	Non-	Pre-treatment tumor	11
(25 July 2017)	node	Deletion positive	treatment		
					
Surgical	Lung	Not	Erlotinib	Post-treatment tumor	11
resection		conducted	administration	Tumor stroma	11
(18 October 2017)				Normal lung	11
				Remission stroma	13

### Optimization of mRNA and DNA extraction and WGA methods for microdissected FFPE tissues

Most of the pathological tissues collected in clinical practice are preserved in FFPE specimens. FFPE specimens are more difficult to extract nucleic acids than fresh-frozen specimens. The decross-linking treatment is necessary for breaking the methylene cross-links formed between nucleic acids and proteins to extract DNA and RNA from FFPE specimens. The decross-linking treatment has to be the optimal condition that minimizes damage to nucleic acids and maximizes the yield of nucleic acids. Since RNA degrades more easily than DNA, the decross-linking condition must be suitable. We optimized decross-linking conditions to extract RNA and DNA simultaneously from the microdissected FFPE tissue. The decross-linking treatment for RNA purification was performed at 85°C for 5 min (26). The number of genes detected in tissue microdissections (TPM > 0) differed by morphological classification (pre-treatment tumor: 6,160 ± 1,340; post-treatment tumor: 9,690 ± 918; normal lung: 6,694 ± 1,472; tumor stroma: 6,262 ± 1,616; remission stroma: 3,908 ± 1,254) (Student’s t-test, statistically significant at values of *P* < 0.001) (**Figures 2B, C**); 99% of the genes detected in the whole tissue section of lung adenocarcinoma after erlotinib administration were also detectable in tissue microdissections (**Figure 2B**). The number of detected genes in tissue microdissections was similar to that in a tissue section (tissue microdissection: 6,571 ± 2,448; whole tissue section: 8,372 ± 1,382) (**Figure 2C**).

The supernatant after mRNA purification was further decross-linked, and DNA was purified using AMPure beads. The optimal decross-linking condition for DNA extraction was evaluated using mouse liver microdissected FFPE tissues. The microdissected FFPE tissues were decross-linked under four different conditions (85°C for 30 min, 90°C for 5 min, 90°C for 10 min, and 90°C for 15 min). We compared the yield of the WGA product of purified DNA. The decross-linking condition at 90°C for 5 min showed the highest yield of WGA product among the four conditions (34.4 ± 3.3 ng/µl at 90°C for 5 min, 9.0 ± 4.3 ng/µl at 90°C for 10 min, 8.9 ± 6.6 ng/µl at 90°C for 15 min, and 4.1 ± 0.2 ng/µl at 85°C for 30 min) (**Supplementary Figure S2A**). Using this procedure, we established a workflow for the simultaneous extraction of mRNA and DNA from the same tissue microdissection.

We detected EGFR ex19del from the microdissected FFPE tissue using our DNA extraction technique. The WGA is necessary for gene mutation analysis from a small amount of DNA. Amplification bias is likely to occur during WGA in FFPE specimens, because nucleic acids are more degraded in FFPE specimens than in fresh-frozen specimens. In this study, we evaluated two WGA methods (PCR-based WGA: GenomePlex kit and multiple displacement amplification [MDA]-based WGA: REPLI-g kit) regarding the attribution rate to the reference genome and uniformity of coverage. The attribution rate to the reference genome was higher in the PCR-based WGA (36.7 ± 5.8%) than that in the MDA-based method (16.0 ± 23.1%) for the microdissected FFPE tissue (**Supplementary Figure S2B**). We evaluated the whole genome sequence coverage in the Lorenz curve’s cumulative percentage of sequence reads. The PCR-based WGA showed a Lorenz curve closer to the diagonal than the MDA method, indicating a higher uniformity of coverage (**Supplementary Figure S2C**). We used the PCR-based WGA to analyze FFPE specimens of lung adenocarcinoma with the above results. The PCR-based WGA products of the microdissected FFPE tissue met the requirement for gene mutation detection (≤ 10 ng) as described in the Regulations for the Handling of Tissue Specimens for Genomic Medicine (The Japanese Society of Pathology) (30) (**Supplementary Figure S2D**). To detect gene mutation, a portion of the WGA products was used for PCR amplification of the EGFR ex19del region. The PCR products were compared by length using electrophoresis to determine negative or positive gene mutation (**Figure 2D**). The tissue microdissection of the normal lung showed only one peak (103 bp), indicating a negative gene mutation. In contrast, positive control (H1650 extracted DNA) and the tissue microdissection of the pre-treatment tumor showed a short peak (86 or 89 bp) indicating EGFR ex19del positivity with the peak (103 bp) indicating the presence of wild type.

### Site-specific gene expression analysis of lung adenocarcinoma

**Figure 3:**
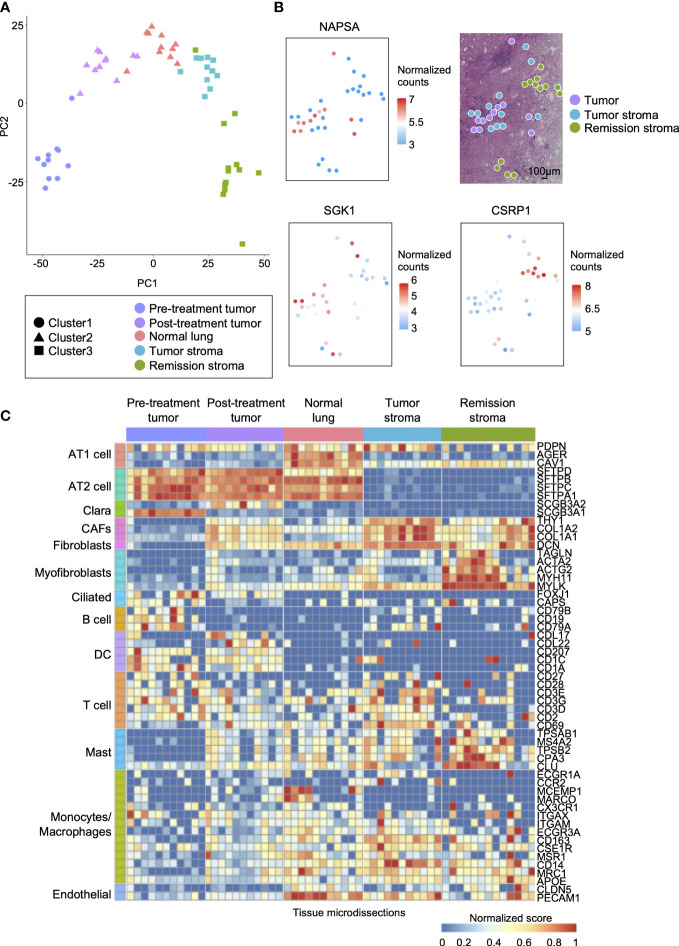
Spatial comparison of transcriptome and EGFR driver gene mutation in lung adenocarcinoma. **(A)** The specific distribution of tissue microdissections in the classification of treatment or histology in PCA. Each shape represents a result of hierarchical clustering using the top 40 genes in average expression level. **(B)** The spatial distribution of site-specific genes in lung adenocarcinoma after erlotinib administration (NAPSA, tumor site-specific gene; SGK1, tumor stroma site-specific gene; CSRP1, stroma in remission site-specific gene). **(C)** Heatmap of marker genes within cell types of lung adenocarcinoma (AT1 cell: PDPN, AGER, and CAV1; AT2 cell: SETPD, SETPB, SETPC, and SETPA1; Clara: SCGB3A1, and SCGB3A2; CAFs: THY1, and COL1A1, and CAL1A2; Fibroblasts: DCN; Myofibroblasts: TAGLN, ACTA2, ACTG2, MYH11, and MYLK; Ciliated: FOXJ1 and CAPS; B cell: CD19, CD79A, and CD79B; DC: CCL17, CCL22, CD207, CD1C, and CD1A; T cell: CD2, CD27, CD28, CD3D, CD3E, CD3G, and CD69; Mast: CPA3, CLU, TPSAB1, TPSB2, and MS4A2; Monocytes/Macrophages: ECGR1A, CCR2, MCFMP1, MARCO, CX3CR1, ITGAX, ITGAM, FCGR3A, CD163, CSF1R, MSR1, CD14, MRC1, and APOE; Endothelial: CLDN5 and PECAM1).

Tumors are composed of a variety of cells. Gene mutations and gene expression of cells vary according to spatial location and histology, even within the same tissue (31, 32). We performed RNA-seq on the tissue microdissections collected from five regions (pre-treatment tumor, post-treatment tumor, normal lung, tumor stroma, and remission stroma). First, we compared the gene expression profiles of each tissue microdissection. Based on the hierarchical clustering analysis of the top 40 most expressed genes, the tissue microdissections were classified into three clusters: pre-treatment tumor, post-treatment lung tissue (post-treatment tumor and normal lung), and post-treatment stroma (tumor stroma and remission stroma) (**Figure 3A** and **Supplementary Figure S3**). The result showed that the expression profiles differed according to histological classification and erlotinib administration.

Next, we analyzed differentially expressed genes among the histologically distinct regions (post-treatment tumor, normal lung, tumor stroma, and remission stroma) in the lung adenocarcinoma after erlotinib administration (*p* < 0.05, |log_2_foldchange| > 1). We found that 3,730 genes including lung adenocarcinoma biomarkers NPC2 (33) and NAPSA (34) were differentially expressed between normal lung (*n* = 11) and post-treatment tumor (*n* = 11) (**Supplementary Table S5** and **Supplementary Figure S4A**). NAPSA was highly expressed in all tissue microdissections of post-treatment tumors regardless of the sampling location in the tissue sections. It was low in tumor stroma and remission stroma around post-treatment tumor, indicating a specific tumor marker (**Figure 3B**).

Between the tumor stroma (*n* = 11) and the remission stroma (*n* = 13), classified as cluster 3 stroma, 2,371 genes were extracted (**Supplementary Table S6**). In the tumor stroma, genes associated with immunodeficiency states favor cancer cell growth (35), and genes related to PD-L1 expression and PD-1 checkpoints, such as T cell receptor signaling pathway and allograft rejection, were predominantly expressed (**Supplementary Figure S4B**). In the comparison of tumor stroma with adjacent remission stroma, SGK1 (36), associated with lung lymph node metastasis, distant metastasis, and poor prognosis in NSCLC, was predominantly expressed in the tumor stroma, whereas the tumor suppressor gene CSRP1 (37) was predominantly expressed in the remission stroma (**Figure 3B**). The tumor stroma and the remission stroma were histologically similar, but differentially expressed genes had different effects on the tumor.

We inferred cell types contained in each tissue microdissection from the marker gene expression of lung constituent cells, including AT cells, Clara cells, fibroblasts, myofibroblasts, ciliated cells, and immune cells. The subtypes of immune cells included B cells, DC (Dendritic cells), T cells, mast cells, monocytes/macrophages, and endothelial cells (**Figure 3C**). AT1 and AT2 cell markers (38, 39) and endothelial cell markers were highly expressed in normal lung. In the pre-and post-treatment tumors, the marker of cells considered the origin of adenocarcinomas, such as AT2 composing the alveolar space and Clara cells composing the bronchioles (40), were highly expressed. Cancer-associated fibroblast (CAF) markers in cancer growth and invasion (41) were highly expressed in tumor stroma. Conversely, myofibroblast markers (42), fibroblast activated and differentiated during wound healing, involved in tissue repair in collaboration with other stroma, and mast cell markers were highly expressed in the remission stroma. Most immune cell markers were not specific to histological classification. Their expression levels differed among tissue microdissections within the same histological classification.

### Analysis of gene mutations and expression in lung adenocarcinoma before and after erlotinib administration

**Figure 4:**
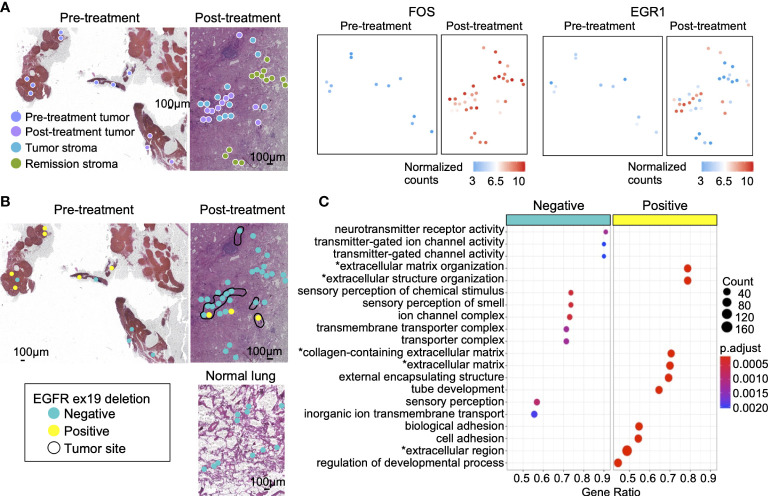
Gene mutations and gene expression in lung adenocarcinoma. **(A)** The genes highly expressed in post-treatment tumors compared with pre-treatment tumors. The oncogenic transcription factor FOS was highly expressed in post-treatment tumors, tumor stroma, and remission stroma. EGR1, a tumor suppressor gene, was highly expressed only in post-treatment tumors. **(B)** The spatial distribution of the EGFR ex19del. Left: tumor in pre-treatment lung lymph node; Right: tumor in post-treatment lung adenocarcinoma. Bottom: normal lung in post-treatment lung adenocarcinoma. The area surrounded by the black line is the tumor. **(C)** The enriched pathways in EGFR ex19del- negative or EGFR ex19del-positive tissue microdissections. Asterisk(*) related to ECM.

We examined changes in gene expression and mutations between tumors before and after erlotinib administration. Between pre-treatment tumor (*n* = 11) and post-treatment tumor (*n* = 11), 3,809 genes with variable expression were detected (**Supplementary Table S7**). Cancer cell invasion and proliferation genes were predominantly expressed in post-treatment tumors, such as the IL-17 signaling pathway and ECM-receptor interaction (43, 44). Moreover, the PI3K-Akt signaling pathway (45) downstream of EGFR, activated in drug-resistant cancer cells with loss of EGFR driver gene mutation, was highly expressed in post-treatment tumors (**Supplementary Figure S4C**). Previous research has studied the expression level of immediate early genes (IEGs) expressed by cells in response to stress and extracellular stimuli as a prognostic factor in cancer. In some cancers, co-expression of IEGs, such as FOS, JUN, and EGR1, was positively correlated with poor survival (46). Here, we detected the site-specific expression of these genes. The oncogenic transcription factors FOS (**Figure 4C**) and JUN (**Supplementary Figure S5A**), highly expressed in post-treatment tumors, were also upregulated in the stroma (tumor stroma and remission stroma) around post-treatment tumors. In contrast, EGR1, a tumor suppressor gene, was predominantly expressed in post-treatment tumors, and its expression was lower in the stroma around the post-treatment tumor (**Figure 4C**).

The lung lymph node collected before treatment has detected EGFR ex19del in gene mutation testing. In this study, EGFR ex19del was detected in 6/11 (54%) of pre-treatment tumors, 2/11 (18%) of post-treatment tumors, and 1/11 (9%) of tumor stroma. Tissue microdissections positive for EGFR ex19del had no positional specificity. They were scattered on the tissue section (**Figure 4B**). Between EGFR ex19del positive tissue microdissections (*n* = 8) and negative tissue microdissections (*n* = 14) in pre-and post-treatment tumors, 1,341 genes with variable expression were extracted. Most of them were genes related to ECM (**Supplementary Table S8** and **Figure 4C**).

### Interaction between tumor and tumor stroma in the tumor microenvironment

**Figure 5:**
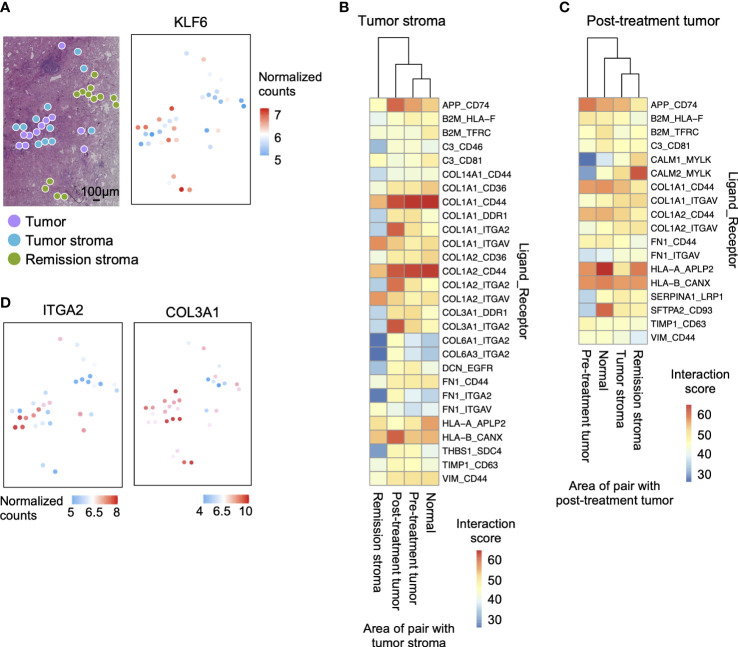
Interaction between tumor and tumor stroma in the tumor microenvironment after erlotinib administration. **(A)** The spatial distribution of the tumor marker gene. KLF6 is more highly expressed in stroma than in the tumor on the tissue section. **(B)** Interaction scores between ligands expressed in tumor stroma and receptors expressed in other regions, showing pairs of tumor stroma and post-treatment tumor interaction scores greater than 35. **(C)** Interaction scores between ligands expressed on post-treatment tumor and receptors expressed in other sites, showing pairs of post-treatment tumor and tumor stroma interaction scores greater than 35. **(D)** The spatial distribution of ligand-receptor pairs related to ECM cell–cell interaction on the tissue sections. The ECM ligand COL3A1 is highly expressed in the tumor stroma, and the ECM receptor ITGA2 is highly expressed in the tumor.

Tumor cells attract fibroblasts, extracellular matrix, and inflammatory cells to form the tumor microenvironment. Those stromal cells interact with tumor cells in ligand-receptor interactions and are involved in tumor growth and invasion. Here, we investigated how tumor and tumor stroma function in cancer survival, growth, and invasion in lung tissue after erlotinib administration. In post-treatment tumors, genes related to drug metabolism-cytochrome P450 were predominantly expressed (**Supplementary Figure S4D**). In tumor stroma, pathways involved in cancer cell invasion and proliferation were activated, such as the TGF-beta signaling pathway (47) and MAPK signaling pathway (**Supplementary Figure S4D**). Most genes considered tumor specific in previous studies (tumor suppressor genes: SOCA3, KLF6, and PTGDS [48–50]; tumor enhancer genes: SLC38A2 [51, 52], DUSP1 [53], and FGF7 [54]) were expressed predominantly in tumor stroma or remission stroma than that in pre-treatment tumors (**Figure 5A** and **Supplementary Figure S5B**).

We then predicted the interactions involved in the invasion and proliferation of tumor tissue in pre-treatment tumors and tumor stroma by the expression levels of ligands and receptors. We calculated the ligand-receptor interaction score (11) by calculating the product of the average expression of the ligand in pre-treatment and tumor stroma and the expression of the receptor in other regions (pre-treatment tumor, post-treatment tumor, normal lung, tumor stroma, and remission stroma). We examined all ligand-receptor pairs involving tumor cells (55, 56) and identified the specifically co-expressed pairs in post-treatment tumors and tumor stroma (**Figures 5B, C** **)**. Stromal cells are the primary source of ligands. Therefore, there were more pairs with high ligand-receptor interaction scores when the tumor stroma was the ligand (**Figure 5B**). Among the interactions involved in tumor invasion and growth, extracellular matrix interaction scores were higher between tumor stroma and post-treatment tumor (tumor stroma ligand: COL1A1, COL1A2, and COL3A1; post-treatment tumor receptor: ITGA2). After that, we mapped the expression level of the pair co-expressed between post-treatment tumors and tumor stroma (COL3A1; ECM ligand and ITGA2; ECM receptor) on the tissue section. ITGA2 was highly expressed in the spots adjacent to the tumor stroma expressing COL3A1 of post-treatment tumors (**Figure 5D**).

## Discussion

We achieved simultaneous extraction of mRNA and DNA from tissue microdissections using two types of magnetic beads. The mRNA purification using oligo dT magnetic beads is an efficient method for RNA-seq analysis of small amounts of RNA (27). It could separate mRNA from other cellular components in tissue microdissections. Here, DNA was purified from the fractions after mRNA purification using carboxyl-coated magnetic beads (AMPure beads XP), commonly used for DNA purification. Pathological specimens are often preserved as FFPE specimens, which are difficult to extract nucleic acids because of the cross-linking between proteins and nucleic acids. Therefore, we established a method of mRNA and DNA extraction method for microdissected FFPE tissues that minimized damage and increased the yield by optimizing decross-linking treatment. The nucleic acids in FFPE specimens are more degraded than fresh-frozen specimens cryopreserved immediately after collection. The MDA-based WGA (REPLI-g kit) is a WGA method using Phi29 polymerase and random primers with a low error insertion rate. It is used for the WGA method with the lowest rate of amplification bias and insertion of artifactual mutations for fresh-frozen specimens (57). However, for FFPE specimens, the PCR-based WGA (GenomePlex kit) showed much lower amplification bias and variant allele frequency (VAF) than the MDA-based WGA (**Supplementary Figure S2**), as shown in a previous study (58). The process fragmented DNA to around 200 bp, which was then PCR amplified to reduce the bias of genome amplification. Using the optimized methods of DNA and mRNA extraction and WGA, we detected about 4,000 to 10,000 expressed genes and EGFR driver gene mutations from tissue microdissections of FFPE specimens stored for four years.

Results from RNA-seq of tissue microdissections showed that gene expression profiles differed according to histological classification. In post-treatment tumors, genes such as FOS, JUNB, COL1A2, and DUSP1 have shown to favor cancer proliferation and invasion (46, 53) and were predominantly expressed compared with pre-treatment tumors. In lung adenocarcinoma after erlotinib administration, bypass pathways that activate cell proliferation and invasion, such as the MAPK signaling pathway (tumor stroma) and PI3K-Akt signaling pathway (post-treatment tumor), were activated. CAFs, a significant component of the tumor stroma, directly interact with tumor cells and induce EMT, contributing to resistance acquisition, cancer invasion, and metastasis (59). Our data showed tumor stroma predominantly expressed genes that promote cancer growth, such as genes indicating immunodeficiency and genes involved in cell-matrix interaction, compared with remission stroma. SLC38A2, DUSP1, and FGF7 expressed predominantly in tumor sites than that in normal lung in previous studies (51, 52); however, they were expressed predominantly in the tumor stroma in this study. These results revealed that even in the same tumor tissue, different microstructures have different properties in cancer. After drug administration, it is desirable to analyze at the microdissection level to study tissue diversity. Currently, there are no prognostic factors established for neoadjuvant therapy. Some cases estimated the efficacy prediction by the tumor cells remaining in the tissues after treatment (60). Based on the results of this study, it might be necessary to evaluate not only the tumor cells remaining after drug administration but also the tumor stroma.

The detection of EGFR driver mutation is necessary for predicting drug efficacy, but its low detection sensitivity is a problem (61). It is difficult to collect the tumor site area during biopsy collection specifically. We could not genetically test some specimens, because the number of tumor cells in the biopsy tissue is too small to detect. In such cases, re-biopsy burdened the patient. This study detected EGFR ex19del from microdissected FFPE tissues. This method is considered more sensitive than detecting gene mutations in a single section, because it can detect gene mutations from the tissue microdissection collected from the specific area gathering tumor cells. However, some tissue microdissections of biopsy with positive EGFR mutation turned out to be negative for the mutation (**Figure 4A**). These results reiterated the difficulty in ensuring the accuracy of clinical diagnoses, such as selecting the biopsy collection site and assuring results by collecting multiple sites. Tumors are composed of cells with diverse gene mutations. If gene mutation testing is performed from tissue microdissections with high cancer cell density, detecting gene mutations in multiple tissue microdissections within the same tissue is necessary. It would be performed gene mutation testing without overlooking drug-sensitive tumors. In this study, the loss of EGFR mutations was confirmed in the tumor sites after EGFR-TKI administration. It might relate to the prognosis, worsening of the disease condition, and acquisition of resistance to the administered drugs (45). However, this study analyzed only one specimen. It is necessary to verify the results in other specimens in future research.

We established a method to simultaneously extract mRNA and DNA from tissue microdissections of lung adenocarcinoma stored as FFPE specimens for 4 years. Our method enables simultaneous detection of gene expression profiles and gene mutation with spatial information of biological tissues. We performed RNA-seq and EGFR driver gene mutation detection in the tumor microenvironment of lung adenocarcinoma before and after EGFR-TKI administration. Using RNA-seq analysis, we detected genes with variable expression among tissue microdissections with different treatment courses and histological classifications. Our spatial gene expression technique revealed that the expression distribution of invasive and proliferative genes of cancer cells differed among adjacent regions (post-treatment tumor, tumor stroma, and remission stroma) on the same tissue section. Activation of cell proliferation pathways and higher expression of cancer-related genes were observed in the tumor stroma, positively affecting cancer invasion, survival, and proliferation, than that in post-treatment tumors. Gene mutation analysis detected different positive rates of EGFR ex19del in lung adenocarcinoma before and after erlotinib administration. There was no area specificity in EGFR ex19del-positive tissue microdissections. Using our method, we detected diverse micro-regions in gene expression and mutation in lung adenocarcinoma after drug administration and different statements of cancer-related genes in tumors and tumor stroma. This method could be applied to pathological specimens stored in medical institutions for a long time to construct a database. It would significantly contribute to identifying molecular markers that can determine drug sensitivity and predict prognosis.

## Data availability statement

The datasets presented in this study can be found in online repositories. The names of the repository/repositories and accession number(s) can be found below: https://www.ncbi.nlm.nih.gov/, PRJNA820591 https://www.ncbi.nlm.nih.gov/, PRJNA820562.

## Ethics statement

The studies involving human participants were reviewed and approved by the regional ethical committee at Juntendo University School of Medicine. The patients/participants provided their written informed consent to participate in this study. The animal study was reviewed and approved by the Committee for Animal Experimentation of the School of Science and Engineering at Waseda University.

## Author contributions

MY, MH, HM, and HT conceived and designed the experiments. MY, MH, HM, KA, and HK conducted the experiments, collected the data, and analysed the results. KT and KS collected clinical data. MY, MH, HM, and HT wrote the manuscript. All authors contributed to the article and approved the submitted version.

## Funding

This research was supported by Platform Project for Supporting Drug Discovery and Life Science Research [Basis for Supporting Innovative Drug Discovery and Life Science Research (BINDS)] from AMED under Grant Number JP21am0101104 and Research Support Project for Life Science and Drug Discovery (BINDS) under Grant Number JP22ama121055.

## Acknowledgments

We thank Naoko Suzuki and Chikako Sakanashi for providing technical support. Also, we would like to thank the Human Genome Center (University of Tokyo, Tokyo, Japan) for providing the supercomputing resources.

## Conflict of interest

HK is a founder and shareholder of Frontier Biosystems, Inc., which provides the microdissection punching system.

The remaining authors declare that the research was conducted in the absence of any commercial or financial relationships that could be construed as a potential conflict of interest.

## Publisher’s note

All claims expressed in this article are solely those of the authors and do not necessarily represent those of their affiliated organizations, or those of the publisher, the editors and the reviewers. Any product that may be evaluated in this article, or claim that may be made by its manufacturer, is not guaranteed or endorsed by the publisher.
