# Supplementary material for: Integrated spatial analysis of gene mutation and gene expression for understanding tumor diversity in formalin-fixed paraffin-embedded lung adenocarcinoma
Source: Front Oncol. 2022 Nov 24;12:936190. doi: 10.3389/fonc.2022.936190 (PMC9731154; doi:10.3389/fonc.2022.936190)
Supplement: Supplementary file 1 [file DataSheet_1.pdf]

Supplementary Material

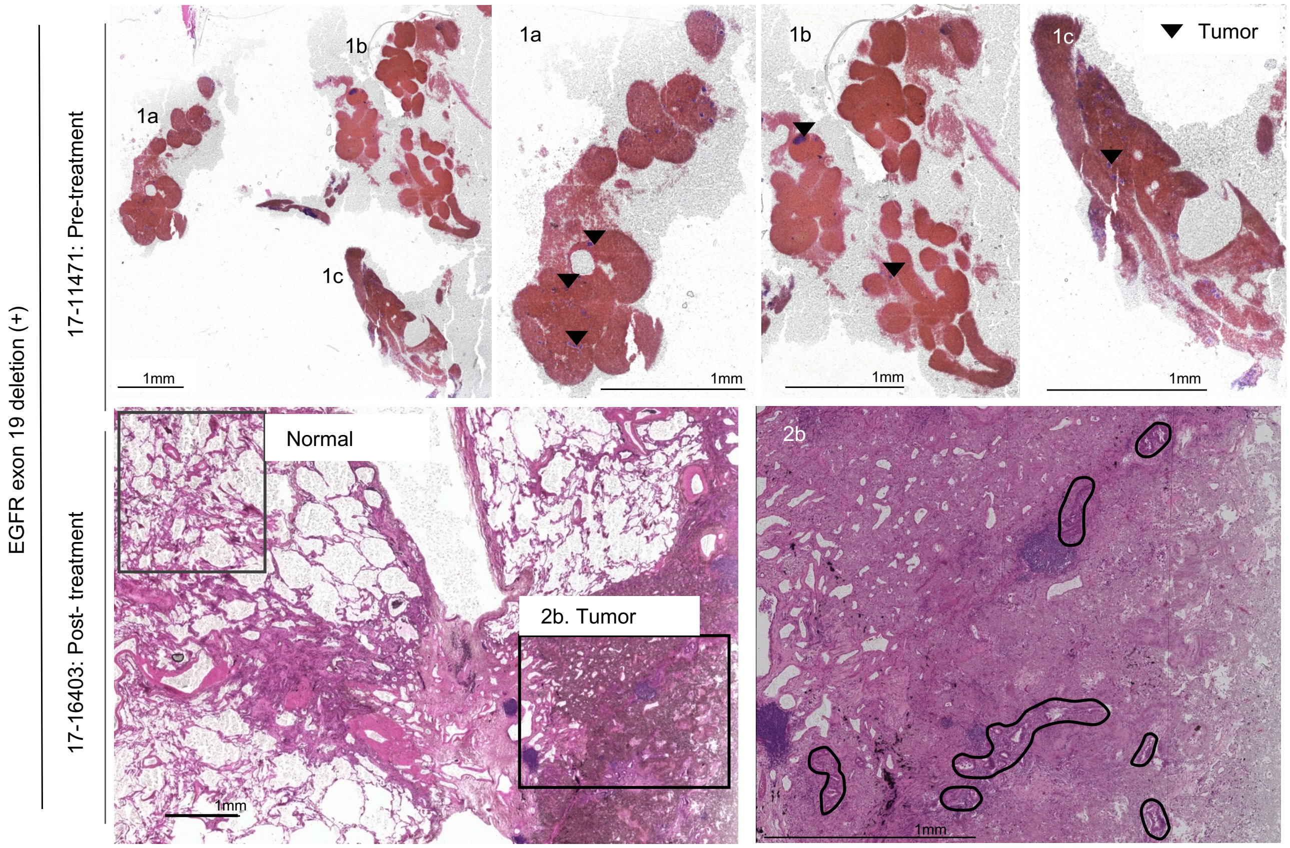

Supplementary Figure 1. Morphological classification of HE-stained images of tissue sections in tumor and non-tumor areas.

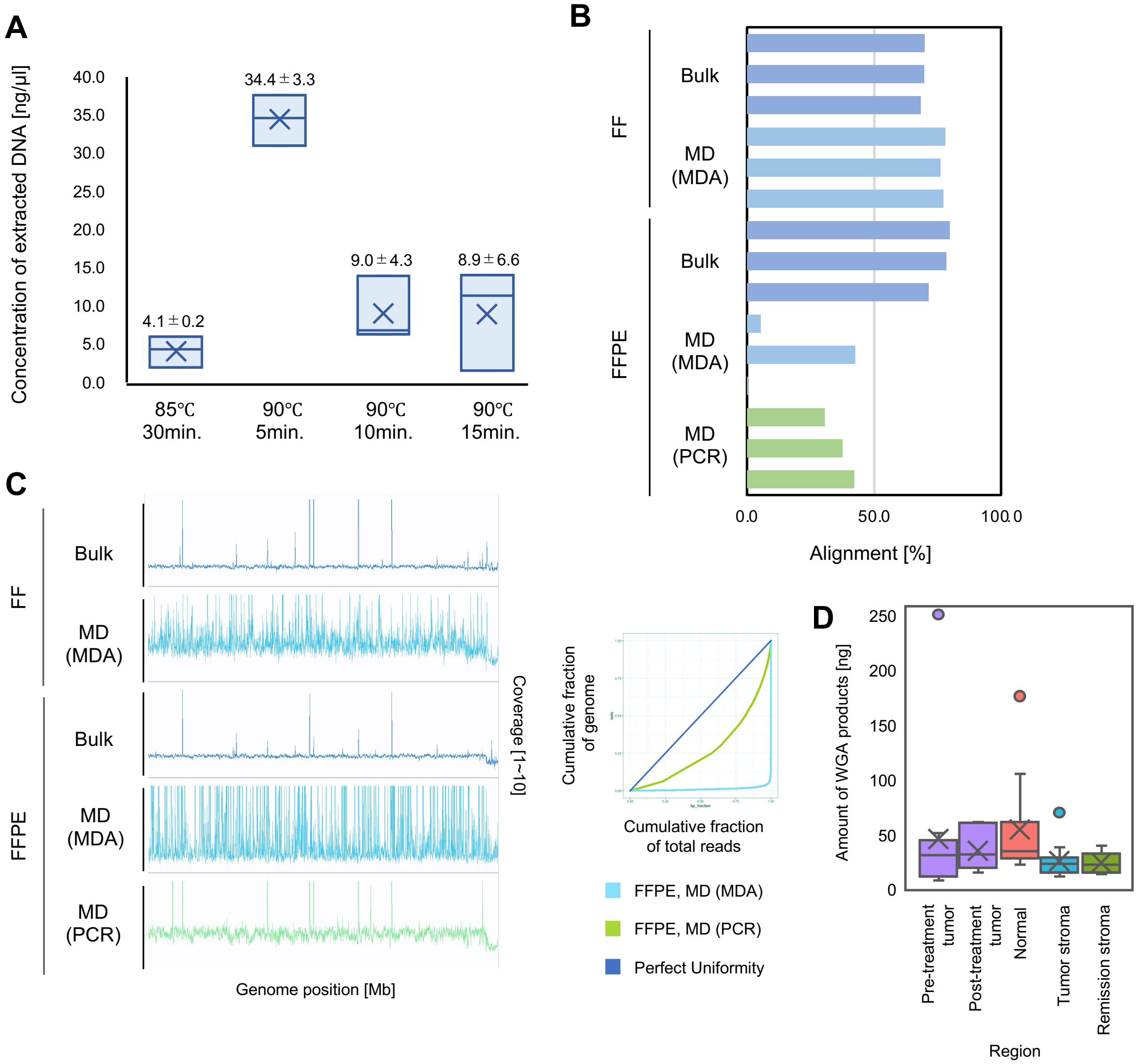

Supplementary Figure 2. Evaluation of DNA extraction and whole genome amplification methods from mouse liver micro-dissected tissues. (a) Comparison of concentration of extracted DNA by different de-crosslinking conditions. Mouse liver micro-dissected FFPE tissues with a diameter of 100 μm were subjected to proteolytic cytolysis using proteolytic enzymes followed by decrosslinking treatment under four conditions (85 ℃ for 30 min, 90 ℃ for 5 min, 90 ℃ for 10 min, and 90 ℃ for 15 min) to determine the respective concentrations after whole genome amplification. (b) Alignment rate of sequence reads to the reference genome. Mouse liver micro-dissected FFPE tissues were subjected to proteolytic cytolysis using proteolytic enzymes followed by decrosslinking treatment at 90 ℃ for 5 min. and PCR-based or MDA-based whole genome amplification. The tissue section and Fresh Frozen (FF) micro-dissected tissues were used to control. (c) Left: coverage distribution of sequencing read in the whole genome output by the Integrative Genomics Viewer (IGV); Right: Lorenz curve of micro-dissected tissue (MD) amplified the whole genome by MDA-based or PCR based. Lorenz curve shows the cumulative fraction of the total reads covering a given cumulative fraction of the genome. The diagonal line indicates a perfectly uniform coverage, and a significant

deviation from the diagonal line indicates a biased coverage. (d) Amount of WGA product from micro-dissected tissue for each histological classification.

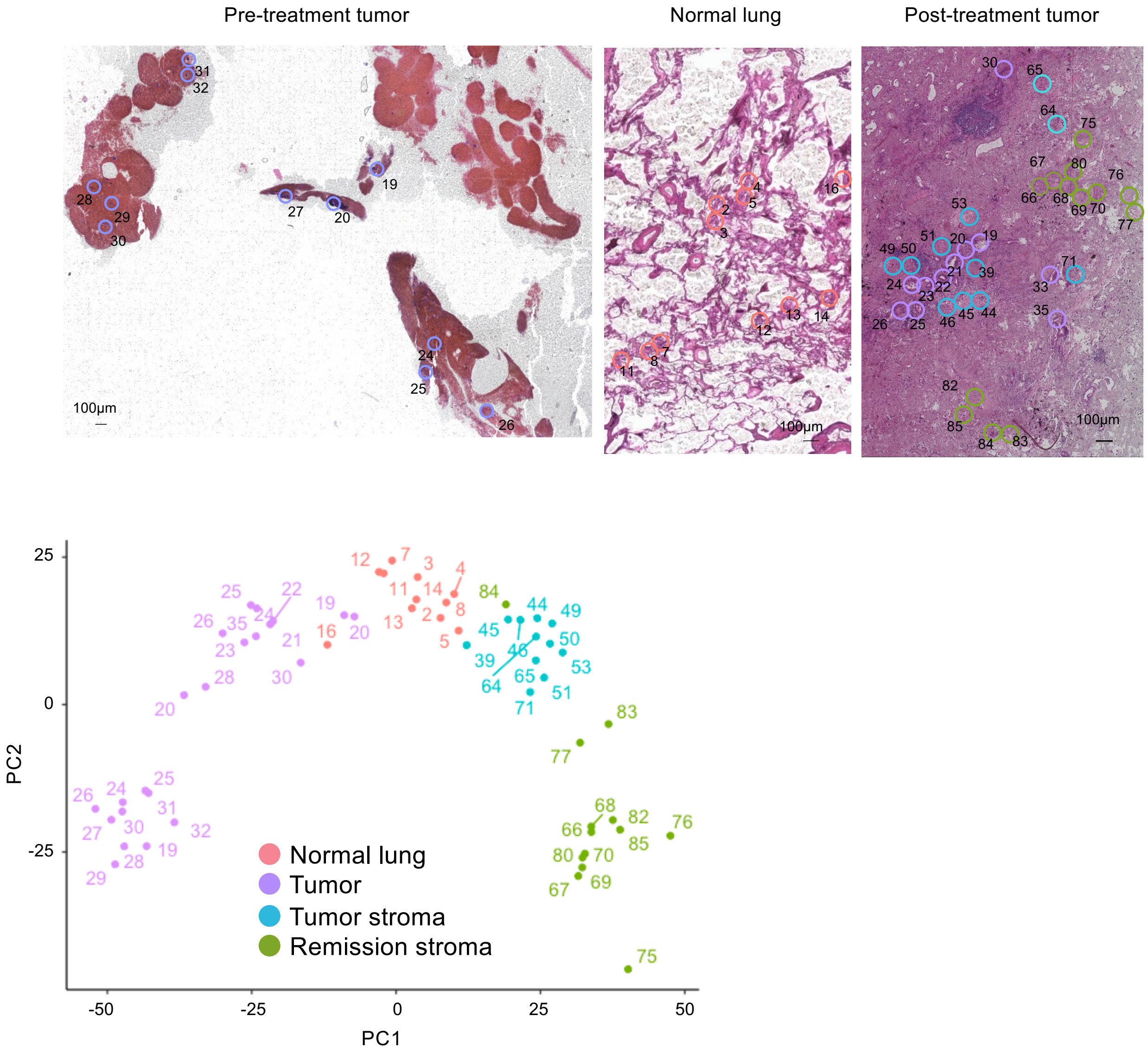

Supplementary Figure 3. Relationship between cluster classification by gene expression profile and sampling location. Tissue section collection location and PCA for each micro-dissected tissue, identified by number. One pre-treatment tumor micro-dissected tissue (number 20) was classified near the post-treatment tumor micro-dissected tissue.

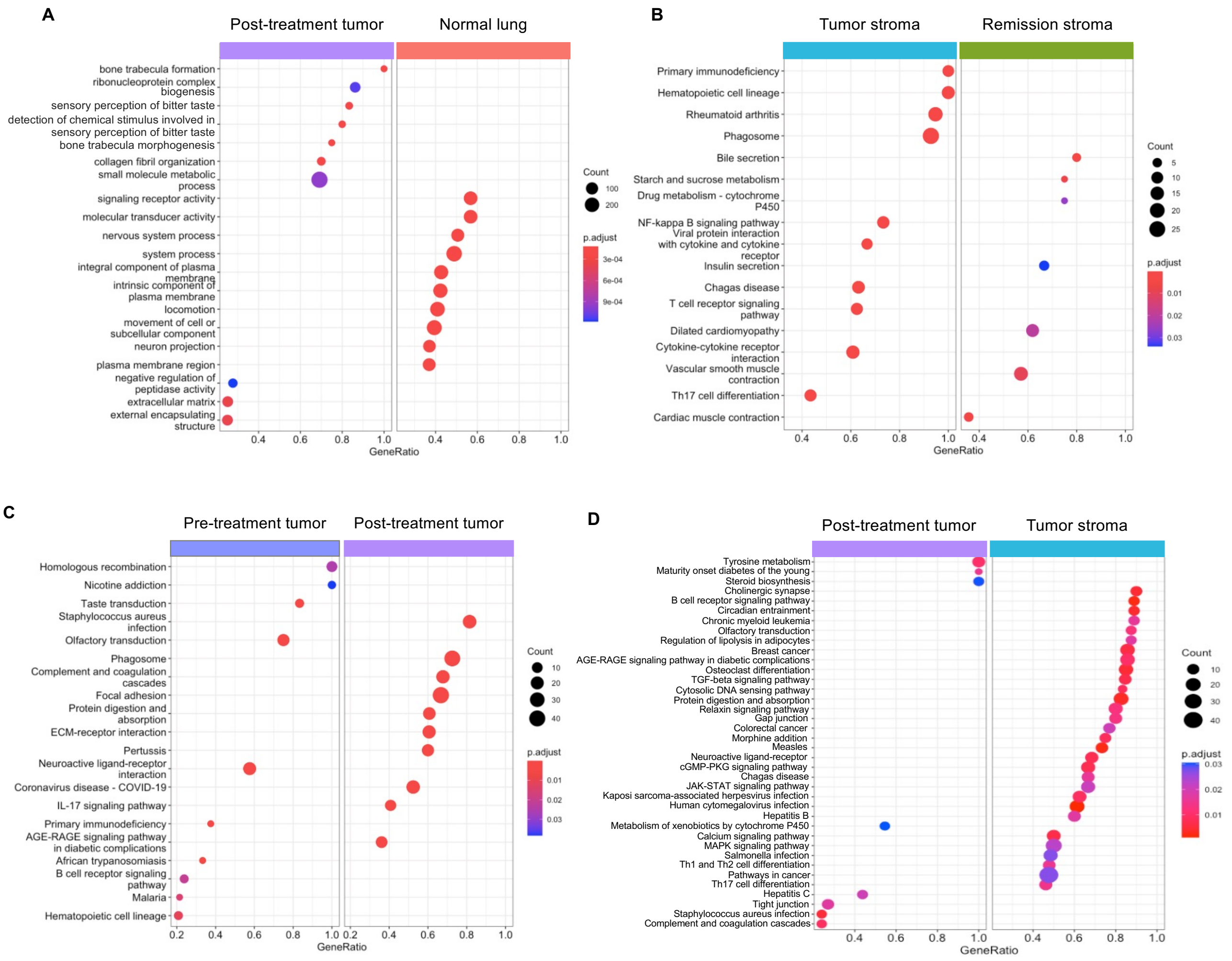

Supplementary Figure 4. Differentially expressed genes. (a) Post-treatment tumor vs. normal lung. (b) Tumor stroma vs stroma in remission. (c) Pre-treatment tumor vs. post-treatment tumor. (d) Post-treatment tumor vs. tumor stroma.

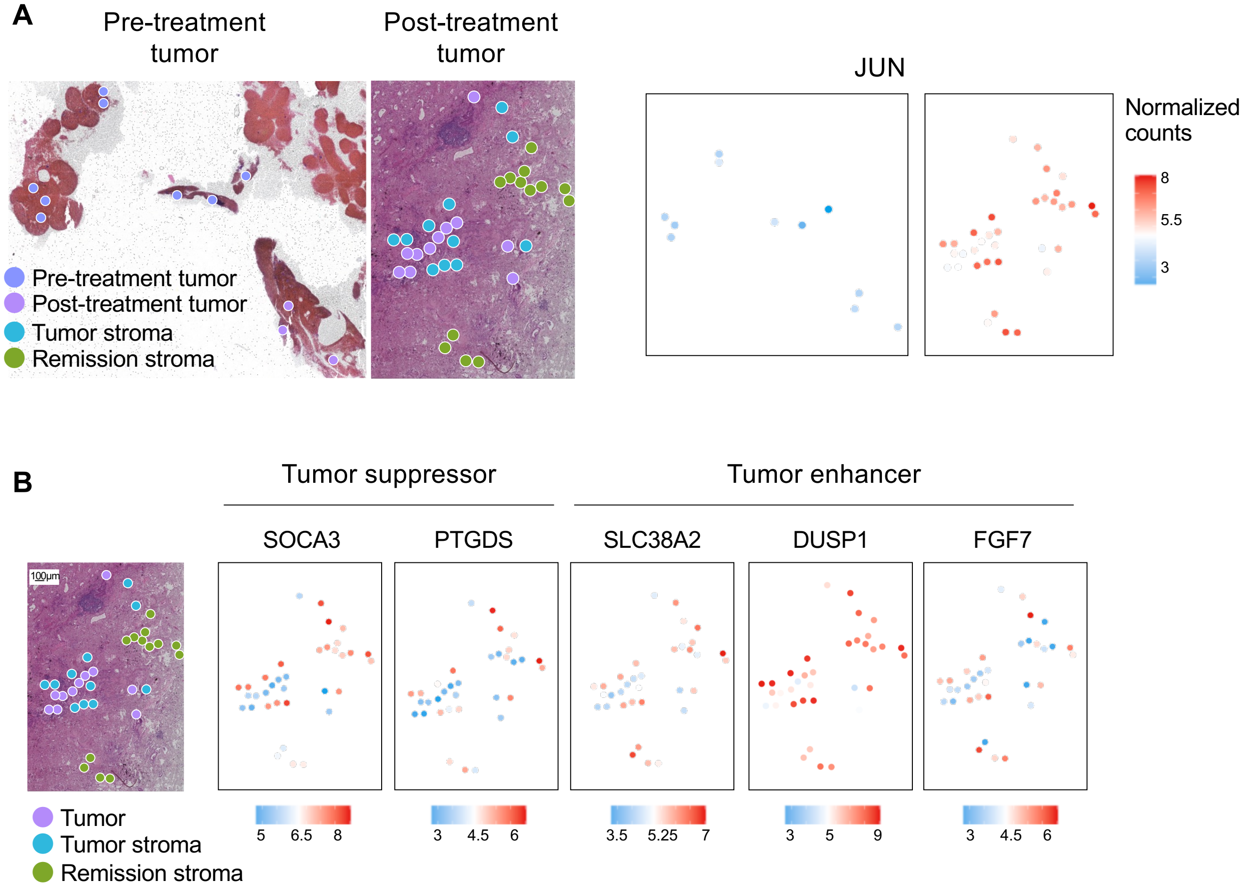

Supplementary Figure 5. (a) Genes highly expressed in post-treatment tumors compared to pre-treatment tumors. The oncogenic transcription factor JUN was highly expressed in post-treatment tumors, tumor stroma, and stroma in remission. (b) The expression distribution of the tumor marker that highly expressed in stroma than in the tumor (T) on the sections. SOCA3 and PTGDS are tumor suppressor genes. SLC38A2, DUPS1, and FGF7 are tumor enhancer genes.
